# Ultrasound visualization of an underestimated structure: the bicipital aponeurosis

**DOI:** 10.1007/s00276-017-1885-0

**Published:** 2017-06-08

**Authors:** M. Konschake, H. Stofferin, B. Moriggl

**Affiliations:** Division of Clinical and Functional Anatomy, Department of Anatomy, Histology and Embryology, Medical University of Innsbruck, Müllerstr. 59, 6020 Innsbruck, Austria

**Keywords:** Bicipital aponeurosis, Lacertus fibrosus, Biceps brachii muscle, Ultrasonography

## Abstract

**Purpose:**

We established a detailed sonographic approach to the bicipital aponeurosis (BA), because different pathologies of this, sometimes underestimated, structure are associated with vascular, neural and muscular lesions; emphasizing its further implementation in routine clinical examinations.

**Methods:**

The BA of 100 volunteers, in sitting position with the elbow lying on a suitable table, was investigated. Patients were aged between 18 and 28 with no history of distal biceps injury. Examination was performed using an 18–6 MHz linear transducer (LA435; system MyLab25 by Esaote, Genoa, Italy) utilizing the highest frequency, scanned in two planes (longitudinal and transverse view). In each proband, scanning was done with and without isometric contraction of the biceps brachii muscle.

**Results:**

The BA was characterized by two clearly distinguishable white lines enveloping a hypoechoic band. In all longitudinal images (plane 1), the lacertus fibrosus was clearly seen arising from the biceps muscle belly, the biceps tendon or the myotendinous junction, respectively. In transverse images (plane 2) the BA spanned the brachial artery and the median nerve in all subjects. In almost all probands (97/100), the BA was best distinguishable during isometric contraction of the biceps muscle.

**Conclusion:**

With the described sonographic approach, it should be feasible to detect alterations and unusual ruptures of the BA. Therefore, we suggest additional BA scanning during clinical examinations of several pathologies, not only for BA augmentation procedures in distal biceps tendon tears.

## Introduction

The biceps brachii muscle (BM) is attached distally to the radial tuberosity via the strong biceps tendon (BT) and to the antebrachial fascia via the bicipital aponeurosis (BA), also known as lacertus fibrosus. As previously described, the BT consists of two distinct portions separated by an endotenon septum and surrounded by a common paratenon, which includes also the BA [5]. The latter may be regarded as the fascial expansion of the BT that finally reaches as far as to the posterior margin of the ulna [7]. Comparable expansions are present at different muscles throughout the body and their common functional significance is force transmission between adjacent muscles and force transmission to non-muscular tissue [13]. In doing so, the BA supports flexion of the elbow on the one hand and, by stabilizing the BT distally, reduces stress concentration at the BT enthesis [9]. The aponeurosis in a broader sense consists of three layers: the thickest, superficial one originates from the anterior radial aspect of the long biceps head, just proximal to the commencement of the distal biceps tendon; it passes distally to the musculotendinous junction of the short head. The rudimentary middle layer acts as mesentery and attaches to the short head; it passes in an ulnar direction to merge anteriorly with the superficial layer. The deep layer originates from the deep radial side of the musculotendinous junction of the long head; it travels in an ulnar direction deep to the tendon of the short biceps head to merge with the other two layers [9]. The merged layers continue distally, superficial to the ulnar flexor muscles, releasing strong fascial adhesions to the ulnar flexor muscles, which tether the aponeurosis. The BA and its continuance encircle the forearm flexors and enforce the antebrachial fascia. Moreover, the BA increases the effectiveness of the BM as a supinator as it tensions the deep antebrachial fascia [3] and acts as a strength strap for increasing the synergy between the biceps brachii muscle and the flexors of wrist and fingers during strong apprehension grip. The greatest power will be achieved in varus position of the wrist due to the contraction of the flexor carpi ulnaris muscle reinforced by the tension of the BA.

The important but often disregarded functional role of the BA is also reflected by the clinical observation that retraction of a ruptured BT is more striking in case the BA is ruptured too [18]. It has also been reported that a ruptured BA may be accompanied by BT-elongation with a weakening of both, elbow flexion and supination [20]. One may hypothesize two patterns of underlying pathogenesis: a previously injured but healed BT with secondary ruptured BA or a primary ruptured BA with secondary elongated BT. Whatever the case, such observations outline the functional importance of the BA by all means.

Despite the well-known difficulties of reliable ultrasound (US) examination of the BT, it has extensively been used to evaluate the tendon’s normal and pathologic status. Quite in contrast and considering the above mentioned functional impact of the BA, it is surprising that we lack reports on US-evaluation of this second BM distal attachment. Reasons may be thinness of this structure and its most superficial location that would equally require procedural skills and excellent high-resolution transducers. This was the reason to establish a sonographic approach to the BA in a cohort of subjects.

## Materials and methods

**Fig. 1:**
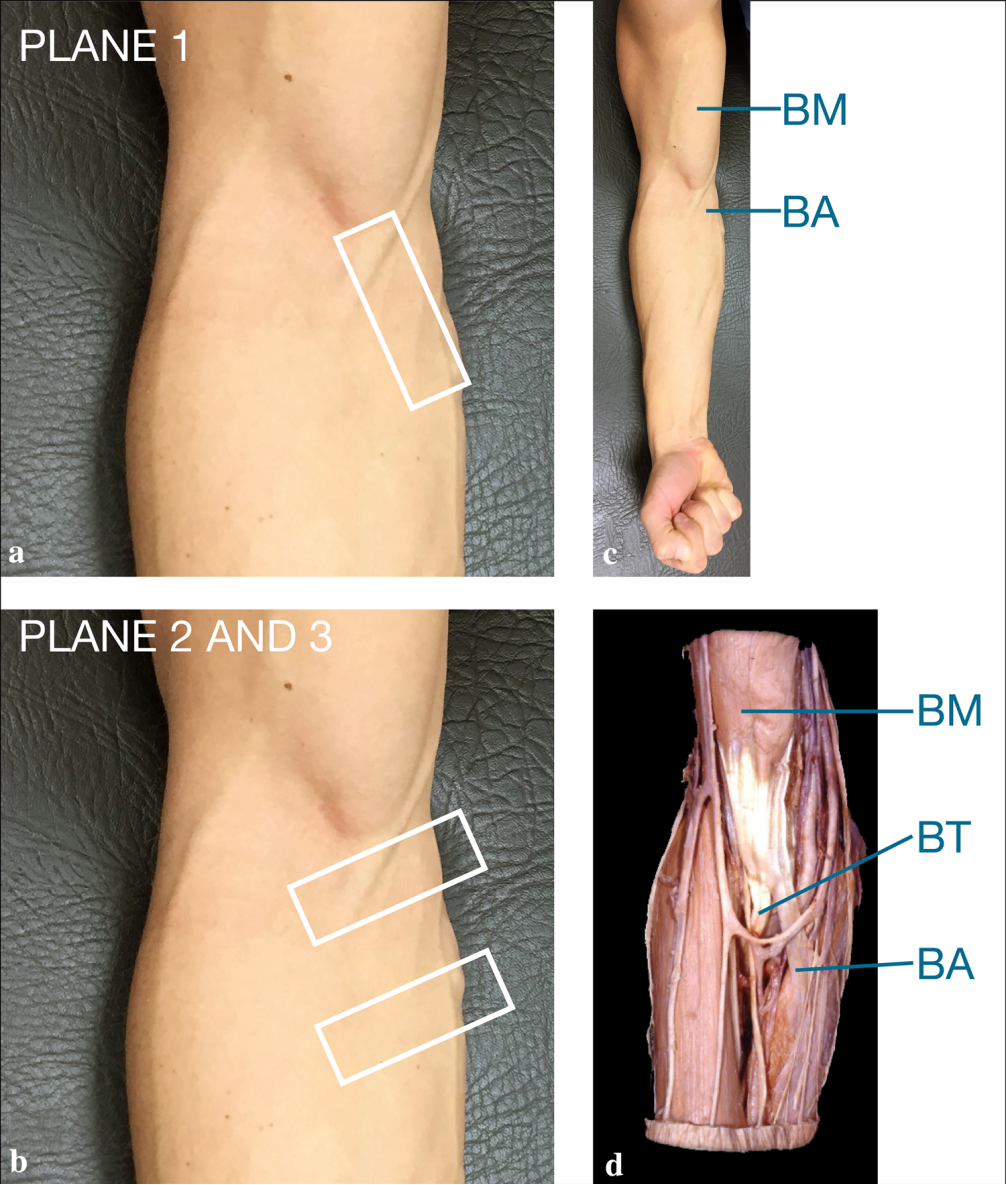
**a** Probe placement for plane 1, longitudinal view: the transducer was placed in line with the assumed aponeurosis’ main bundle, illustrated by the *white rectangle*. **b** Probe placement for plane 2 and 3, transverse views: the probe was turned 90° at two different levels as shown by the *two white rectangles*. **c** Isometric contraction during examination, showing the biceps brachii muscle (BM) and the bicipital aponeurosis (BA). **d** Specimen showing the biceps brachii muscle (BM), the biceps tendon (BT) and the bicipital aponeurosis (BA)

The investigation was performed in 100 volunteers (50 women, 50 men) aged 18–28 (mean age women 22.9 ± 2.3, mean age men 24.3 ± 1.8) according to the Declaration of Helsinki. Exclusion criteria were: trauma or previous operation of the upper limp, obvious aberrance of the normal physiognomy of the upper arm and pre-existing chronic tendon disease or systemic diseases affecting connective tissues. All scans were done using an 18–6 MHz linear transducer (LA435; system MyLab25 by Esaote, Genoa, Italy) utilizing the highest frequency. Participants were sitting and facing the operator with the elbow lying on a suitable table (Fig. 1a, b, c). The BA was scanned in two planes: for the first one (longitudinal view) the transducer was placed in line with the assumed aponeurosis’ main bundle (Fig. 1a), for the second plane (transverse view) the probe was turned 90° at two different levels (Fig. 1b).

**Fig. 2:**
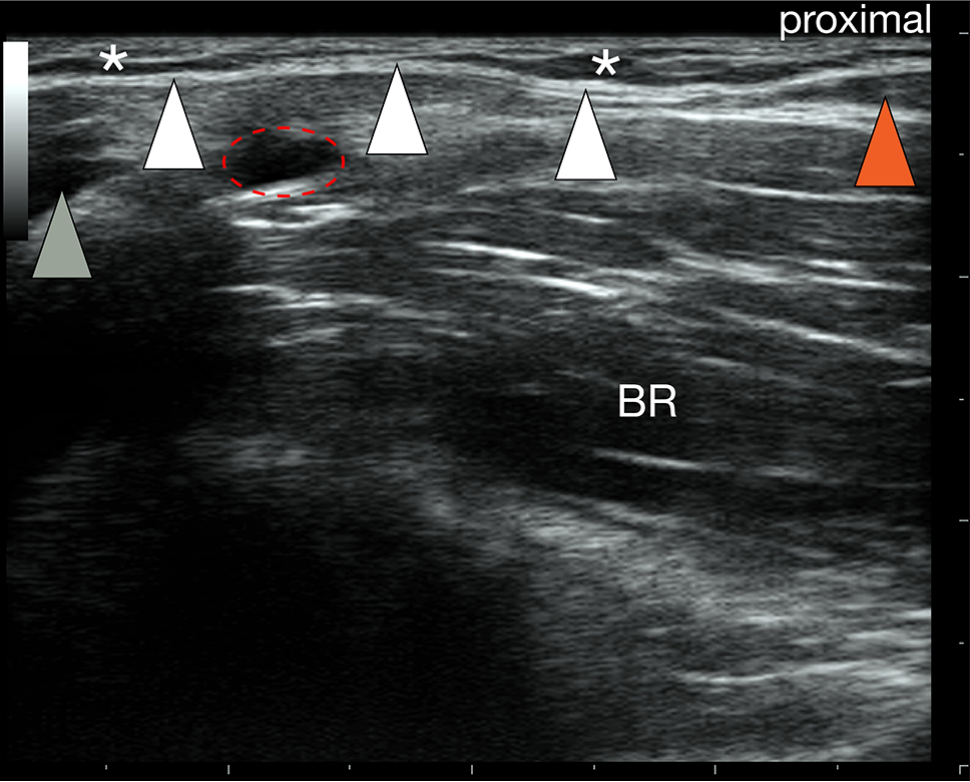
Bicipital aponeurosis (BA) longitudinal view. The BA (*white arrowheads*) is seen as double contour emerging from the myotendineous junction of biceps brachii muscle (*orange arrowhead*), bridging the brachial artery (*red dashed oval*) and connecting to the antebrachial fascia that covers the pronator teres muscle (*grey arrowhead*). Note that the BA is clearly distinguishable from the subcutis (*asterisks*)! *BR* brachialis muscle (color figure online)

**Fig. 3:**
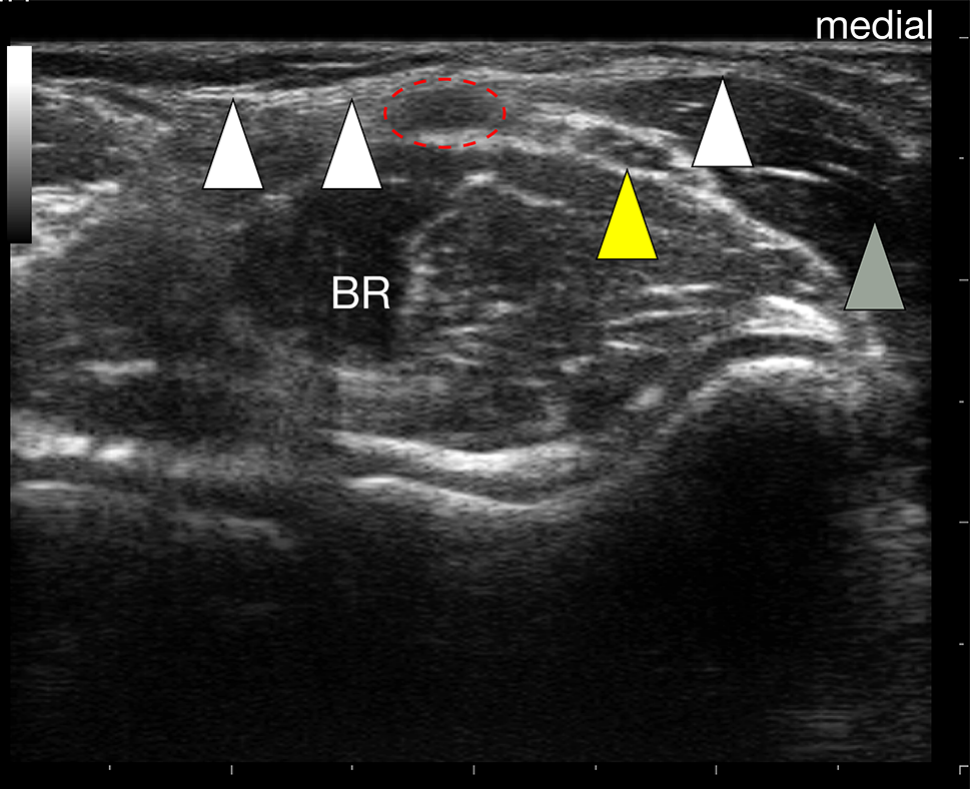
Bicipital aponeurosis (BA) transverse view. The BA (*white arrowheads*) is seen as double contour bridging the brachial artery (*red dashed oval*) and the median nerve (*yellow arrowhead*) before it connects to the antebrachial fascia that covers the pronator teres muscle (*orange arrowhead*). Note that, due to anisotropy, the biceps tendon is not delineated here in contrast to Fig. 4. *BR* brachialis muscle (color figure online)

**Fig. 4:**
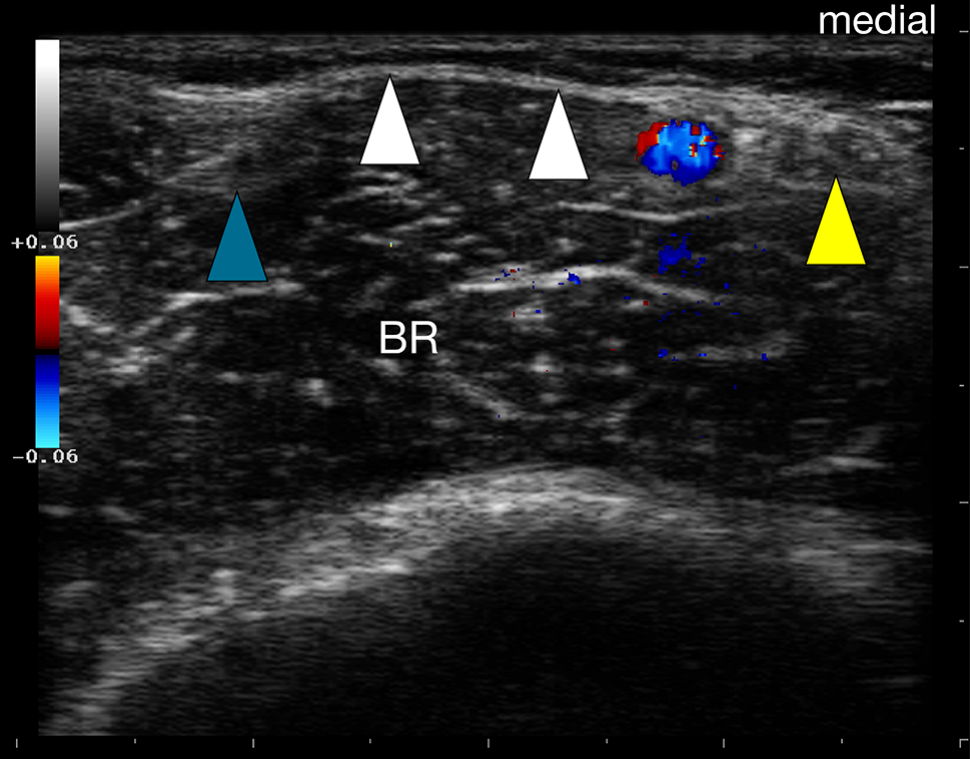
Bicipital aponeurosis (BA) transverse view more proximal compared to Fig. 3. The BA (*white arrowheads*) is seen as double contour bridging the brachial artery (*colored mainly blue*) and the median nerve (*yellow arrowhead*). Note that the BA appears slightly *arched*; the biceps tendon (*blue arrowhead*) is partially seen. *BR* brachialis muscle (color figure online)

To identify the main bundle easily, we palpated the biceps tendon in the antecubital fossa, placed the probe slightly proximally and rotated it towards the ulna. Hence the probe was aligned obliquely: proximally to the myotendinous junction of the BM and distally to the dorsal border of the upper part of the ulna (Fig. 2). After detection of the BA, the brachial artery and the pronator teres muscle, the probe was turned 90 degrees for the second plane at two different levels—both perpendicular to plane one (see and compare Figs. 3, 4). In each proband, scanning was done with and without isometric contraction of the BM (Fig. 1). In both planes, a second image was gained with color coded Duplex sonography to additionally document the brachial artery that regularly runs deep to the BA. Due to the obvious sparseness of the BA (taking the scale of the system as reference it was obvious that thickness was always less than 1 mm), no measurements were taken, because inherent measurement errors would have led to unacceptable pseudo accurateness.

## Results

We could identify the BA in both planes in all subjects, aged 18–28 (mean age women 22.9 ± 2.3, mean age men 24.3 ± 1.8), investigated. The BMI was 22.9 ± 3.0 kg/m^2^, seven probands (two females) were left handed. Only 26 probands (14 females) answered not to be physically active in any way, whereas 23 (10 females) practiced sports involving the upper limb. A total number of 18 probands (8 females) were smokers (female averaged number 14.1 cigarettes per day, males averaged 7.8 cigarettes per day).

The BA was characterized by two clearly distinguishable white lines enveloping a hypoechoic band (Figs. 2, 4). In all longitudinal images (plane 1), the lacertus fibrosus was clearly seen arising from the BM belly, the BT or the myotendinous junction, respectively. Further, bridging the brachial artery and connecting to the antebrachial fascia that covers the pronator teres muscle. Additionally, the BA was clearly distinguishable from the subcutis (Fig. 2). In transverse images (plane 2) the BA spanned the brachial artery (Fig. 4) and the median nerve in all subjects. In almost all probands (97/100), the BA was best distinguishable during isometric contraction of the BM. The two parallel layers of the BA appeared slightly arched and faded into the antebrachial fascia in both planes imaged.

## Discussion

Appropriate experience in scanning the MSK system is a prerequisite for successful elbow imaging. US is still considered an operator-dependent procedure. But it offers advantages over other imaging tools such as magnetic resonance imaging: it is fast, it is economical, has superior spatial resolution and gives the important possibility of dynamic examination [22]. Not long ago imaging of the BA was considered impossible [14], but the rapid technological development of high-resolution and high-quality probes opened the possibility to scan even small and tiny structures. Therefore, we describe a detailed sonographic approach to the lacertus fibrosus, confirmed in 100 volunteers. We found no influence of physical activity level, BMI, smoking or sex on BA visualization feasibility.

For a clinical context, it is worth mentioning that what is illustrated here as the US representation of the BA is in fact the central main part of that flared out BM insertion. Throughout the cohort, visibility of the BA was best during isometric contraction against resistance. This is important as many of the investigations within the MSK system is done both, at rest and dynamically. Previous authors suggested the basilic vein as a good landmark in imaging the BA, at the same time stating that not too much pressure should be exerted, because the vein is easily compressed [8]. This is the very same reason why we dismissed the basilic vein as a landmark. Not till enough pressure is applied, the BA is clearly visible and distinguishable from both the subcutaneous and muscular tissue. Furthermore, the basilic vein exhibits marked topographic variability in the cubital fossa [27]; identifying the vein at its consistent proximal level, tracking it distally and then visualizing the BA seems inefficient. We advocate our technique of placing the probe proximally obliquely from the biceps tendon towards the upper part of the dorsal border of the ulna. Providing a faster and easier way to image the lacertus fibrosus distinctively. We point out that, due to anisotropy, the biceps tendon was not always clearly delineated in transverse images, therefore, constituting an inept landmark. Similarly, a variation of the brachial artery, termed superficial brachial artery, should not misguide the examiner: in 9% it replaces the normal brachial artery and often runs to the forearm anterior to the BA [17]. Identifying the brachial artery deep to the BA is helpful in most cases, but not as exclusive parameter in pinpointing the latter. The examiner is guided much better by the emergence of the BA from the biceps brachii muscle, running subcutaneously and connecting to the antebrachial fascia that covers the pronator teres muscle. The work of Snoeck et al. concentrated on the correlation between anatomical and morphometric variations of the BA with anthropometric and morphometric measurements of the upper limb [23]. They could not demonstrate a significant correlation, but could identify individual characteristics of the BA and described a deep layer ending on the deep surface of the pronator teres muscle, which merges with the neurovascular tract. A finding also confirmed in our ultrasound based study.

Why is US imaging of the BA important? Recently, Fontana et al. suggested and performed BA scanning for autoplastic BA augmentation [11]. Their report concentrated on the surgical technique and its advantages, without describing their approach to BA scanning, but recommending pre-operative ultrasound to evaluate BA integrity, size and shape—emphasizing the importance of BA scanning [11]. With our description at hand, routine implementation of BA scanning for several distinct clinical problems should be feasible and we want to discuss examples for its application:

Ultrasound has a pivotal role in partial tears of the distal biceps attachments, where MRI has difficulties in determining what percentage of the latter is torn. A contiguity of the short biceps head and an intact BA prevents the typical retraction of the muscle belly—complicating a fast diagnosis [25]. This delay may preclude primary repair or lead to chronic weakness in supination and flexion [19, 20]. Early treatment is crucial, because early surgery diminishes complication risks and has better results [26]. Underlining the role of a fast and effective diagnostic tool such as US. Moreover, Landa et al. affirmed the importance of BA imaging in complete distal biceps tendon tears: repair of the lacertus fibrosus increased maximum strength by 55–60% and could prevent long-term loss of strength and range of motion, common in traditional repair of the distal biceps tendon [16]. Repair of the lacertus fibrosus may improve cosmetic outcomes by preventing postoperative pitting in the medial aspect of the antecubital fossa. Therefore, we believe US imaging and evaluation of the BA should be implemented in routine protocols for distal biceps tendons tears. In addition, the BA is involved in other pathologies: one case in the literature described a contribution of the BA in a pronator teres syndrome. The patient suffered from bizonal compression; first by the lacertus fibrosus, second by an isolated abnormal tendon of the brachialis muscle [10].

Another distinct entity is median nerve compression by the BA as a result of partial rupture of the lateral, distal myotendinous junction of the biceps [21]; differentiated from incomplete rupture of the distal biceps insertions, because haematoma, cyst formation or elongation of the distal tendon with proximal muscle bulging are absent [6, 12]. The partial rupture, described by Seitz et al., changes the pull of the biceps with proximal and medial shift of the lacertus fibrosus and subsequently compressing the median nerve underneath the leading edge of the BA [21]. The investigated patients presented with severe anterior arm and proximal forearm pain after sudden severe flexion against a severe counterforce; interestingly without diminished median sensory or motor function. Until establishment of the final diagnosis and successful surgery, all subjects endured months of unsatisfactory treatments and misdiagnoses [21]. A similar occurrence was reported in three patients—without description of the causative injury [24]—and in a 47-year-old guitar player [15]. In addition, compression of the sensory portion of the musculocutaneous nerve can develop: particularly if the nerve emerges from beneath the biceps tendon to assume its subcutaneous position at the elbow crease. The lateral margin of the lacertus fibrosus can exert a compression force as the elbow extends, markedly accentuated by full pronation. Especially after strenuous and repetitive movements [1]. We believe that, in the aforementioned pathologies, routine sonographic imaging of the BA and the adherent structures could have provided a proper diagnosis faster, preventing the reported unsatisfactory treatments and misdiagnoses.

Patients affected by rather uncommon pathologies could also benefit from ultrasound examination of the BA: Biemans presented a case of an athletic young male suffering from claudication-type pain, back then diagnosed with angiography. Muscular hypertrophy and concomitant thickening of the BA resulted in entrapment of the brachial artery, successfully resolved by surgical release of the fascial structure [4]. Bassett 3rd et al. described at last five patients suffering from similar symptoms due to hypertrophied forearm muscles; including localized tenderness over the lacertus fibrosus, cold intolerance, increased pain and obliterated radial pulse during forearm pronation and resisted elbow flexion. Satisfactory results were achieved with surgical decompression of the BA and normal pulses were restored in all cases [2].

## Conclusion

The described sonographic imaging of the BA could provide a fast and cost-effective tool in several different pathologies, not just in BA augmentation procedures: with knowledge of the normal sono-anatomic appearance it should be feasible to detect unusual ruptures and alterations of that somehow neglected part of the BM’s distal attachments, associated with vascular, neural and muscular lesions. We encourage the further implementation of our approach in routine examinations to verify our claim in a clinical setting.
